# A Novel Early Life Stress Model Affects Brain Development and Behavior in Mice

**DOI:** 10.3390/ijms24054688

**Published:** 2023-02-28

**Authors:** Hyun Seung Shin, Soo Min Choi, Seung Hyun Lee, Ha Jung Moon, Eui-Man Jung

**Affiliations:** Department of Molecular Biology, College of Natural Sciences, Pusan National University, Busan 46241, Republic of Korea

**Keywords:** early life stress, behavior, mice, calbindin-D_28k_, parvalbumin

## Abstract

Early life stress (ELS) in developing children has been linked to physical and psychological sequelae in adulthood. In the present study, we investigated the effects of ELS on brain and behavioral development by establishing a novel ELS model that combined the maternal separation paradigm and mesh platform condition. We found that the novel ELS model caused anxiety- and depression-like behaviors and induced social deficits and memory impairment in the offspring of mice. In particular, the novel ELS model induced more enhanced depression-like behavior and memory impairment than the maternal separation model, which is the established ELS model. Furthermore, the novel ELS caused upregulation of arginine vasopressin expression and downregulation of GABAergic interneuron markers, such as parvalbumin (*PV*), vasoactive intestinal peptide, and calbindin-D_28k_ (*CaBP-28k*), in the brains of the mice. Finally, the offspring in the novel ELS model showed a decreased number of cortical PV-, CaBP-28k-positive cells and an increased number of cortical ionized calcium-binding adaptors-positive cells in their brains compared to mice in the established ELS model. Collectively, these results indicated that the novel ELS model induced more negative effects on brain and behavioral development than the established ELS model.

## 1. Introduction

Early life is a period sensitive and vulnerable to environmental influences, such as stress, and the negative influences of early life impact later life [1]. It has been demonstrated that exposure to stress in early life may increase susceptibility to metabolic, cardiovascular, and mental diseases in organisms [2,3]. The experimental model of early life stress (ELS) mimics stresses that make up human adversity, including deprivation, maltreatment, and maternal neglect [4,5]. Researchers using ELS models have shown that ELS is associated with aberrant brain plasticity, leading to memory dysfunction, mood disorders, and neurodegeneration [6,7]. In an ELS model with limited bedding material, mouse pups showed fear memory impairment [8]. In an amyloid-based mouse model of Alzheimer’s disease, the same ELS model was applied with added mesh platform conditions, which promoted amyloid plaque formation and altered neuroinflammatory responses in the hippocampus [9]. The maternal separation model alters the gene expression of glucocorticoid receptors related to the hypothalamus-pituitary-adrenal (HPA) axis in the dentate gyrus of offspring mice [10]. ELS, using the neonatal predator odor exposure paradigm, increased the expression level of long-interspersing element 1, a retrotransposon related to psychiatric disorders, such as schizophrenia and bipolar disorder, and induced social deficits in juvenile mice [11]. In the hippocampus of male offspring mice, the expression of cell proliferation and differentiation markers was reduced by an ELS model in which maternal care was inhibited during the developmental period [12]. Additionally, the maternal separation model alters gene expression related to oligodendrogenesis and immediate early gene expression in the medial prefrontal cortex of postnatal day 15 (P15) mice [13]. Based on these results, previous studies have investigated the impact of ELS on brain health but have only used a single ELS condition, such as maternal separation, maternal care fragmentation, or predator odor exposure. Research on the collective effects of diverse ELS conditions is unclear.

In the postnatal period, maternal care is the most important factor for emotional and neural development in offspring [14]. Maternal care can contribute to the survival and growth of the infant and shape behavioral responses in adulthood [15]. In studies investigating the effect of impaired maternal care on offspring, the maternal separation paradigm has been actively used as an ELS model [16,17,18]. A previous study revealed that maternal separation may induce hyperactivity of the HPA axis, leading to psychiatric disorders, such as depression, in offspring mice [19]. An established ELS model, using the maternal separation paradigm, demonstrated that maternal separation causes anxiety-like behavior and elevated plasma adrenocorticotropic hormone levels in rat offspring [20]. Additionally, long-term maternal separation induces a neuroinflammatory response and reduces the expression of collapsing response mediator protein 2, a neuroprotective mediator, in the brains of offspring mice [21].

Currently, the effects of ELS on neurodevelopment are unclear. Thus, to further investigate the influence of ELS on the brains of offspring mice, and to manufacture a suitable ELS model for in-depth research on neurodevelopment, we established a novel ELS model in which the maternal separation paradigm and mesh platform condition were combined. We hypothesized that this model would lead to more negative effects on neurodevelopment in offspring mice than the established maternal separation ELS model. In addition, we focused on changes in behavior and alterations in brain cell distribution in the novel ELS model because previous studies reported that ELS disrupted normal behavior and the balance of cell distribution in the brain [22,23,24]. From our results, we propose that the novel ELS model is more suitable than the established ELS model in studies investigating the impact of ELS on brain development and behavior because of its enhanced negative effect on brain development and behavior in the offspring mice.

## 2. Results

### 2.1. Novel ELS Model Induces Anxiety- and Depression-like Behavior in Offspring Mice

**Figure 1:**
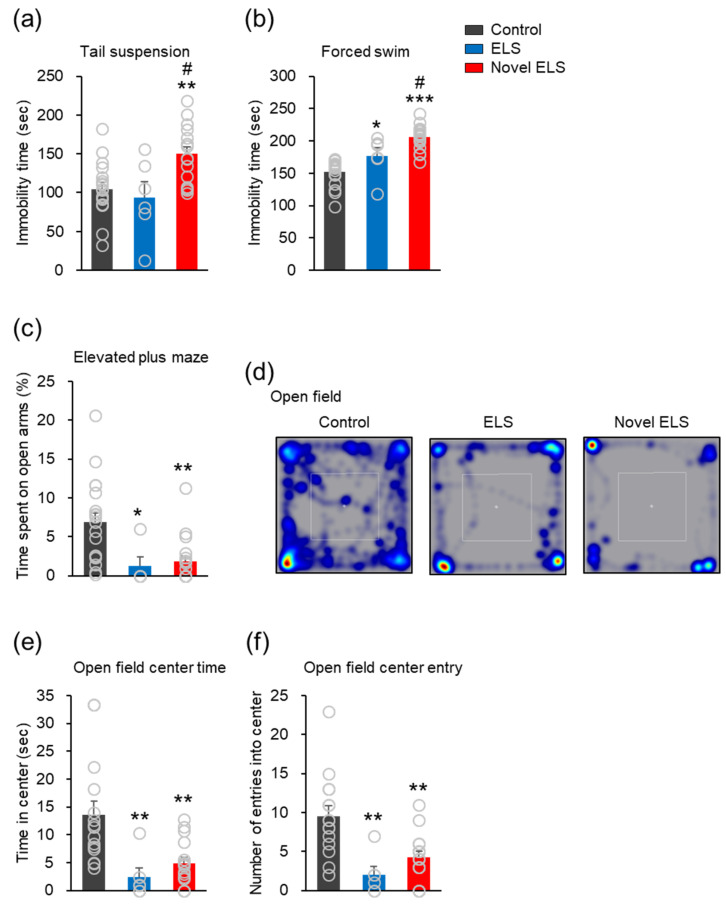
Novel ELS model provokes anxiety- and depression-like behavior in the offspring mice. (**a**) Tail suspension test. (**b**) Forced swimming test. Statistical significance was determined using one-way ANOVA, with Bonferroni’s correction. * *p* < 0.05 and ** *p* < 0.01 vs. vehicle, *** *p* < 0.001 vs. vehicle, # *p* < 0.05 established ELS vs. novel ELS. (**c**) Elevated plus-maze test. Statistical significance was determined using one-way ANOVA, with Bonferroni’s correction. * *p* < 0.05 and ** *p* < 0.01 vs. vehicle. (**d**) Representative images of mouse movement in the open-field test. (**e**) Time spent in the center. (**f**) Number of entries into the center. Statistical significance was determined using one-way ANOVA, with Bonferroni’s correction. ** *p* < 0.01 vs. vehicle. Data represent mean ± SEM.

Tail suspension and forced swimming tests were performed to assess whether the established and novel ELS models induced depression-like behavior in the offspring mice. In the tail suspension test, there was no significant difference in immobility time between the control and established ELS groups (Figure 1a). The novel ELS group displayed significantly higher immobility times than the control and established ELS groups (*F*_2,37_ = 7.818, *p* = 0.0015; control: 18 mice (11 males, 7 females), established ELS: 6 mice (3 males, 3 females), and novel ELS: 16 mice (9 males, 7 females); Figure 1a). In the forced swimming test, all ELS groups showed markedly higher immobility times than the control group. However, the immobility times in the novel ELS group were significantly higher than those in the control and established ELS groups (*F*_2,42_ = 33.86, *p* < 0.0001; control: 20 mice (8 males, 12 females), established ELS: 6 mice (3 males, 3 females), and novel ELS: 19 mice (8 males, 11 females); Figure 1b). These results indicated that the novel ELS model induced more severe depression-like behavior in the offspring mice than the established ELS model. To analyze anxiety-like behavior, an elevated plus-maze test (reflected by the percent time in the open arms) and an open-field test (reflected by the time spent in the center zone) were performed. First, the results of the elevated plus-maze test showed that the percent time in the open arms (*F*_2,39_ = 7.865, *p* = 0.0014) were significantly lower in the established and novel ELS groups than that in the control group (control: 19 mice (9 males, 10 females), established ELS: 5 mice (3 males, 2 females), and novel ELS: 18 mice (8 males, 10 females); Figure 1c). However, there was no significant difference in the results of the elevated plus-maze test between the established and novel ELS groups (Figure 1c). In the open-field test, the established ELS and novel ELS groups displayed significantly lower amounts of time spent (*F*_2,34_ = 8.798, *p* = 0.0008) and number of entries into the center area (*F*_2,34_ = 10.22, *p* = 0.0003) than the control group (control: 15 mice (12 males, 3 females), established ELS: 6 mice (3 males, 3 females), novel ELS: 16 mice (8 males, 8 females); Figure 1d–f). The results of the open-field test were not significantly different between the established and novel ELS groups (Figure 1d–f). These results indicate that the novel ELS model induced anxiety-like behavior in the offspring mice.

### 2.2. Novel ELS Model Induces Deficiency of Social Behavior in the Offspring Mice

**Figure 2:**
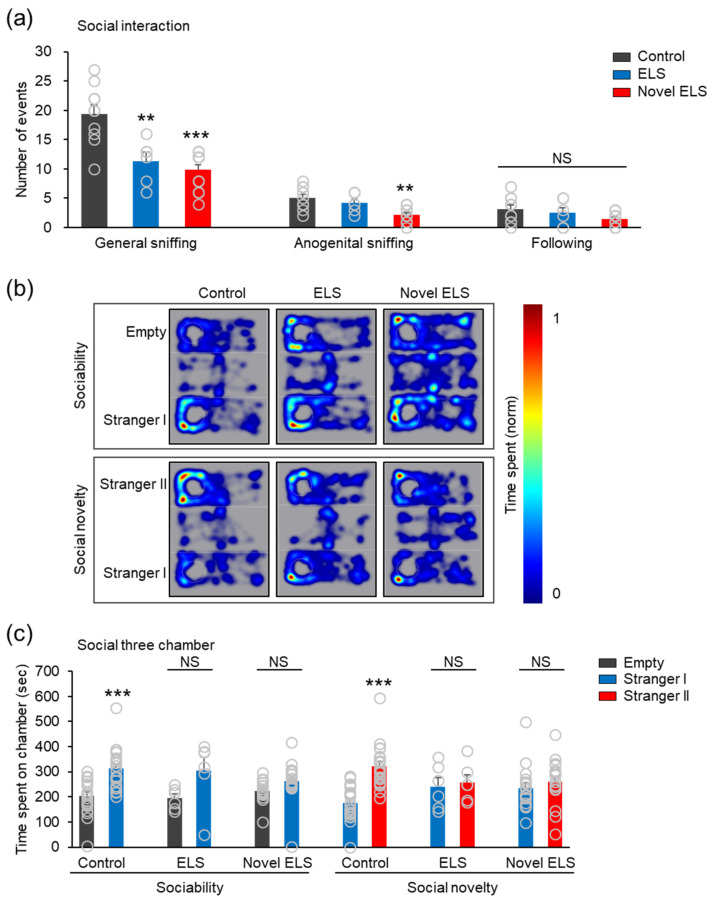
Novel ELS model impairs social behavior. (**a**) Social interaction test. Statistical significance was determined using one-way ANOVA, with Bonferroni’s correction. ** *p* < 0.01 and *** *p* < 0.001 vs. vehicle. NS, no significance. (**b**) Representative heat-map images in the three-chamber test for elucidation of the procedure of sociability and social novelty sessions. (**c**) Three-chamber test. Statistical significance was determined between the times spent in the empty chamber and that with Stranger 1, or times spent with Strangers 1 and 2, for each group and condition, using a two-tailed Student’s *t*-test. *** *p* < 0.001 vs. opposite chamber. Data represent mean ± SEM. NS, no significance.

A social interaction test was performed to evaluate the social interaction behavior of each group. In the social interaction test, there was a lower level of interaction observed in the established and novel ELS groups compared to that in the control group (general sniffing: *F*_2,25_ = 16.41, *p* < 0.0001; anogenital sniffing: *F*_2,25_ = 8.949, *p* = 0.0012) (control: 10 mice (6 males, 4 females), established ELS: 6 mice (3 males, 3 females), and novel ELS: 12 mice (5 males, 7 females); Figure 2a). These results demonstrated that the established and novel ELS models negatively alter social behavior in the offspring mice. Next, we used a three-chamber test to assess social interaction behavior and discrimination of social novelty in each group. First, the mice in each group explored the three-chamber apparatus in which an unfamiliar mouse existed in one of the side chambers (Figure 2b). In the sociability test, while the control group spent more time in the chamber containing the unfamiliar mouse (S1) than in the empty chamber, there was no significant difference in the time spent in the chamber containing the unfamiliar mouse (S1) and the time spent in the empty chamber between the established ELS and novel ELS groups (control; Empty = 204.52 ± 15.28, Stranger I = 313.39 ± 17.61, (*t_40_* = 4.668, *p* < 0.0001); established ELS; Empty = 194.53 ± 17.58, Stranger I = 303.69 ± 53.43, (*t_10_* = 1.941, *p* = 0.081); and novel ELS; Empty = 224.16 ± 10.41, Stranger I = 263.34 ± 19.88, (*t_32_* = 1.746, *p* = 0.0904)) (control: 21 mice (12 males, 9 females), established ELS: 6 mice (3 males, 3 females), and novel ELS: 17 mice (9 males, 8 females); Figure 2c). In the social novelty test, while the control group spent more time in a novel chamber (S2) than in the familiar chamber (S1), the established and novel ELS groups spent almost a similar amount of time in the two chambers (control; Stranger I = 176.09 ± 15.06, Stranger II = 321.47 ± 18.65, (*t_40_* = 6.065, *p* < 0.0001); established ELS; Stranger I = 240.31 ± 36.23, Stranger II = 257.04 ± 30.94, (*t_10_* = 0.3512, *p* = 0.7327); and novel ELS; Stranger I = 235.12 ± 21.31, Stranger II = 260.24 ± 22.37, (*t_32_* = 0.8128, *p* = 0.4223) (control: 21 mice (12 males, 9 females), established ELS: 6 mice (3 males, 3 females), and novel ELS: 17 mice (9 males, 8 females); Figure 2c). These results demonstrated that the novel ELS model induces social behavioral deficits in the offspring mice.

### 2.3. Novel ELS Model Impairs the Cognitive Ability of the Offspring Mice

**Figure 3:**
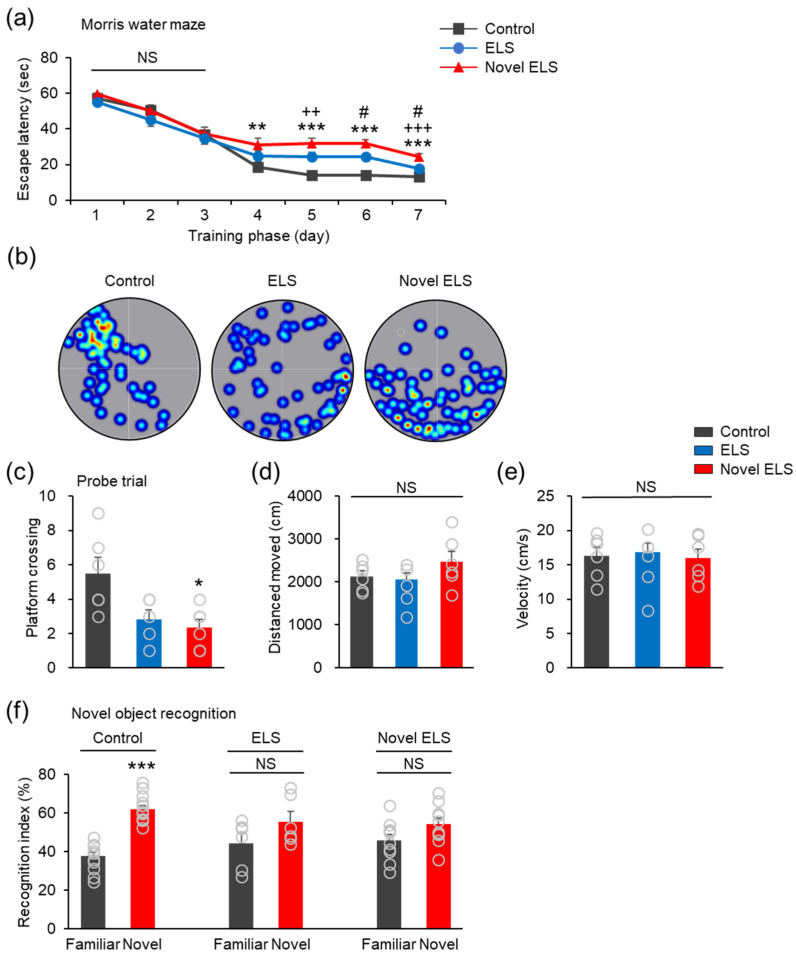
Novel ELS impairs learning and recognition memory in the offspring mice. (**a**) Morris water maze training phase. Statistical significance was determined using one-way ANOVA, with Bonferroni’s correction. ** *p* < 0.01 and *** *p* < 0.001 vs. control, # *p* < 0.05 established ELS vs. novel ELS, ++ *p* < 0.01 and +++ *p* < 0.001 control vs. novel ELS. NS, no significance. (**b**) Representative swim paths of the control, established ELS, and novel ELS groups during a probe trial after training. (**c**–**e**) Morris water maze probe trial. Statistical significance was determined using one-way ANOVA, with Bonferroni’s correction. * *p* < 0.05 vs. control. NS, no significance. (**f**) Novel object recognition test. Statistical significance was determined using a two-tailed Student’s *t*-test. *** *p* < 0.001 familiar vs. novel object. NS, no significance.

Initially, the Morris water maze test was performed to assess the spatial learning and memory ability of each group. There was no significant difference in the time taken to arrive at the hidden platform in all groups during the first 3 days of the training phase (day 1: control = 57.10 ± 0.76, established ELS = 55 ± 1.77, novel ELS = 59.52 ± 0.46; day 2: control = 50.48 ± 2.83, established ELS = 45.25 ± 3.87, novel ELS = 50.18 ± 3.10; day 3: control = 36.82 ± 4.11, established ELS = 34.8 ± 3.33, novel ELS = 37.14 ± 3.79; Figure 3a). The average escape latency was significantly higher in the established and novel ELS groups than in the control group during training days 5 and 7 and between training days 4–7, respectively. The novel ELS group exhibited a markedly higher average escape latency, compared to that in the established ELS group, on training days 6 and 7 (day 4: control = 18.5 ± 2.52, established ELS = 24.75 ± 3.05, novel ELS = 31.25 ± 3.43 (*F*_2,77_ = 5.51, *p* = 0.0058); day 5: control = 14.14 ± 1.43, established ELS = 24.58 ± 2.34, novel ELS = 31.96 ± 2.67 (*F*_2,77_ = 17.36, *p* < 0.0001); day 6: control = 14.14 ± 1.36, established ELS = 24.58 ± 1.15, novel ELS = 31.96 ± 2.04 (*F*_2,77_ = 12.78, *p* < 0.0001); day 7: control = 13.21 ± 0.65, established ELS = 17.75 ± 1.13, novel ELS = 24.36 ± 1.90 (*F*_2,77_ = 25.65, *p* < 0.0001); control: 6 mice (4 males, 2 females), established ELS: 5 mice (3 males, 2 females), and novel ELS: 6 mice (3 males, 3 females); Figure 3a). After the training phase, the platform was removed from the pool for probe testing. There was no significant difference in the crossing number of the area where the platform was previously located between the established ELS and control groups, whereas the novel ELS group exhibited a slightly lower value for the same than the control group (*F*_2,14_ = 6.091, *p* = 0.0125; control: 6 mice (4 males, 2 females), established ELS: 5 mice (3 males, 2 females), and novel ELS: 6 mice (3 males, 3 females); Figure 3c). The distance moved and velocity did not differ significantly between all groups (Figure 3d,e). Representative images of the swim track of all groups revealed that the novel ELS group showed lower proximity to the platform quadrant than the control group (Figure 3b).

We analyzed the recognition memory of each group using the novel object test. The mice were allowed to explore two identical objects, and 6 h later, one of the two objects was replaced by a new novel object. The control group exhibited greater approach time and proximity to the novel object than the familiar object (control; familiar object = 37.90 ± 1.95, control; novel object = 62.10 ± 1.95, (*t_24_* = 8.796, *p* < 0.0001)), but the established ELS and novel ELS groups displayed no difference in approach time and proximity between the familiar and novel objects (established ELS: familiar object = 44.35 ± 5.08, established ELS: novel object = 55.65 ± 5.08, (*t_10_* = 1.573, *p* = 0.1468); novel ELS: familiar object = 45.85 ± 3.08, novel ELS: novel object = 54.15 ± 3.08, (*t_18_* = 1.816, *p* = 0.0861)) (control: 13 mice (7 males, 6 females), established ELS: 6 mice (3 males, 3 females), novel ELS: 10 mice (6 males, 4 females); Figure 3f). Taken together, these results indicate that the novel ELS model impaired spatial learning and recognition memory in the offspring mice and induced more severe memory dysfunction than the established ELS model.

### 2.4. Novel ELS Affects the Growth of Offspring Mice in the Early Stage and the Expression of Genes Associated with Stress and GABAergic Interneurons

**Figure 4:**
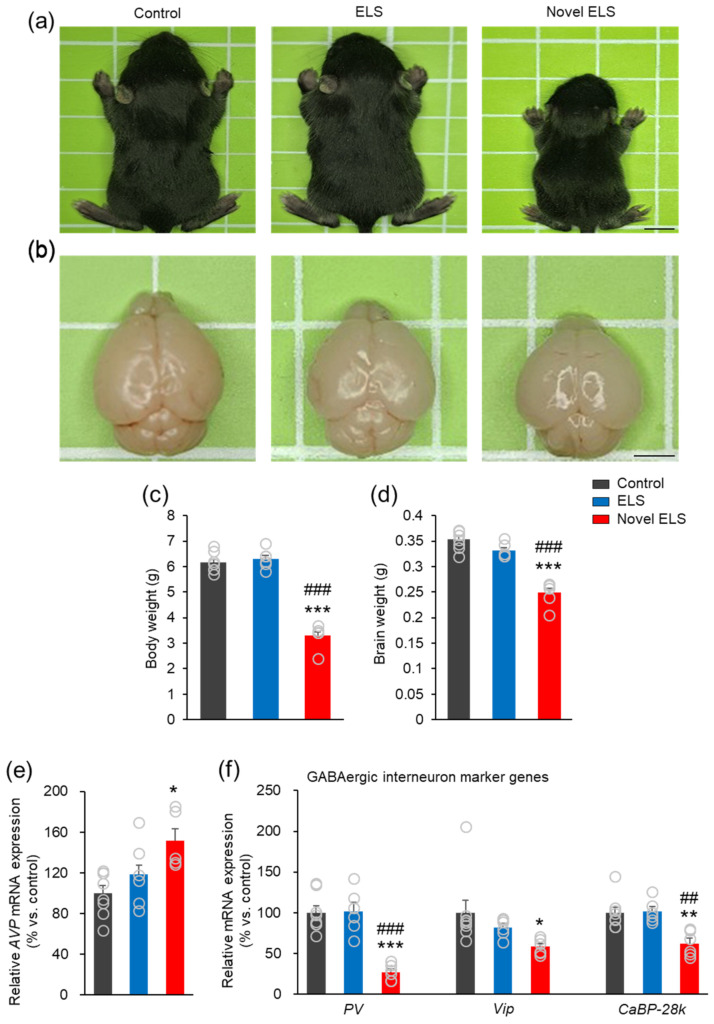
Novel ELS inhibits mouse growth and alters gene expression related to stress and GABAergic interneurons. (**a**,**b**) Representative image of the postnatal day 14 (P14) mouse body and brain for the control, established ELS, and novel ELS groups. (**c**,**d**) P14 offspring mouse body and brain weight. Scale bar = 5 mm (body image) and 3 mm (brain image). Statistical significance was determined using one-way ANOVA, with Bonferroni’s correction. *** *p* < 0.001 vs. vehicle, ### *p* < 0.001 established ELS vs. novel ELS. (**e**,**f**) Quantification analysis of *AVP*, GABAergic interneuron marker gene expression in the P14 offspring mice whole brains through quantitative real-time PCR. Statistical significance was determined using one-way ANOVA, with Bonferroni’s correction. Gene expression was normalized using the mRNA content of GAPDH. * *p* < 0.05 and ** *p* < 0.01 vs. vehicle, *** *p* < 0.001 vs. vehicle, ## *p* < 0.01 and ### *p* < 0.001 established ELS vs. novel ELS. Data represent mean ± SEM.

We estimated the effects of all ELS models on the growth of the body and the brain of early-stage offspring mice. The novel ELS group showed a more significant reduction of body and brain weight on P14 than the control and established ELS groups (body weight: *F*_2,17_ = 113.9, *p* < 0.0001; brain weight: *F*_2,17_ = 53.01, *p* = 0.0012) (control: 6 mice (4 males, 2 females), established ELS: 5 mice (3 males, 2 females), and novel ELS: 6 mice (3 males, 3 females); Figure 4a–d). However, there were no marked differences in body and brain weight in P112 offspring mice for all groups (control: 7 mice (3 males, 4 females), established ELS: 4 mice (2 males, 2 females), and novel ELS: 8 mice (4 males, 4 females); Figure S1a–d). These results indicate that the novel ELS model delays normal brain development in the early stage, but does not persist in the adult stage. It is known that ELS induces the expression of the stress hormone arginine vasopressin (AVP) and corticotropin-releasing hormone, leading to HPA axis activation [25,26]. Additionally, it was reported that ELS disturbs the function of the inhibitory GABAergic interneuron in the brain development stage [23,27]. In this study, we evaluated the effects of the novel ELS model on the expression of genes associated with stress and GABAergic interneurons in brains of P14 offspring mice. First, *AVP* expression increased significantly in the novel ELS group compared to that in the control group (*F*_2,17_ = 5.72, *p* = 0.0134; control: 8 mice (5 males, 3 females), established ELS: 6 mice (3 males, 3 females), and novel ELS: 6 mice (2 males, 4 females); Figure 4e). Moreover, the expression of GABAergic interneuron marker genes, parvalbumin (*PV*) and calbindin-D_28k_ (*CaBP-28k*), decreased significantly in the novel ELS group compared with that in the control and established ELS groups, and vasoactive intestinal peptide (*Vip*) expression also markedly decreased in the novel ELS group, more than that in the control group (*Pavlb*: *F*_2,17_ = 24.52, *p* < 0.0001; *Vip*: *F*_2,17_ = 3.527, *p* = 0.0523; *CaBP-28k*: *F*_2,17_ = 10.71, *p* = 0.001) (control: 8 mice (5 males, 3 females), established ELS: 6 mice (3 males, 3 females), and novel ELS: 6 mice (2 males, 4 females); Figure 4f). These results suggest that the novel ELS model induces a more severe stress response and has a more negative impact on GABAergic interneuron development than the established ELS model in the early stages of life.

### 2.5. Novel ELS Model Alters the Number of Cortical GABAergic Interneuron Subpopulations and Microglial Cell Populations in Adult ELS Mice

**Figure 5:**
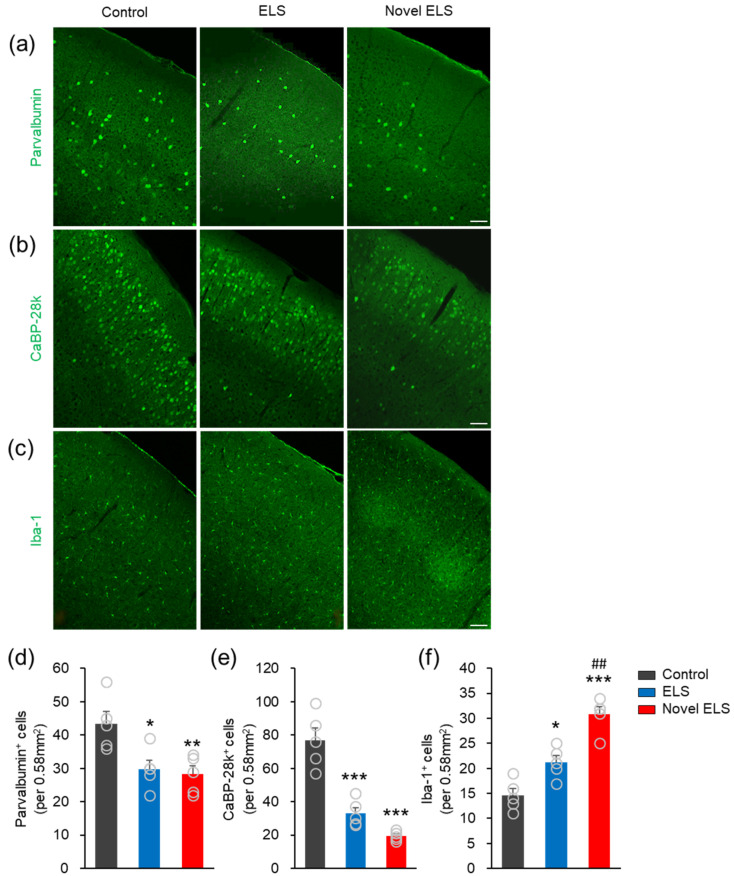
Novel ELS changes protein levels related to the cortical GABAergic interneuron and microglia in offspring mice brains. (**a**–**c**) For immunostaining of coronal sections in the P112 mouse cortex, antibodies against specific GABAergic interneuron markers PV and CaBP-28k (green), and microglia marker Iba-1 (green), were used. (**d**–**f**) Quantitative analysis of (**a**–**c**). Statistical significance was determined using one-way ANOVA, with Bonferroni’s correction. * *p* < 0.05 and ** *p* < 0.01 vs. vehicle, *** *p* < 0.001 vs. vehicle, ## *p* < 0.01 established ELS vs. novel ELS. Data represent mean ± SEM. Scale bar: 100 μm.

Previous studies have reported that the ELS model may alter a number of cortical GABAergic interneuron subpopulations and microglial cell populations in the offspring’s brains [28,29]. In this study, we investigated the changes in the densities of cortical PV^+^, CaBP-28k^+^, and Iba-1^+^ cells in the brains of P112 offspring mice, using established ELS and novel ELS models. The cortical PV^+^ cell densities in the brains of P112 offspring from the established ELS and novel ELS groups were significantly lower than those in the brains of P112 offspring from the control group (*F*_2,12_ = 8.761, *p* = 0.0045; 5 photographs of 0.58 mm^2^ cortical layer in mice (3 male, 2 female) for each group; Figure 5a,d). The cortical CaBP-28k^+^ cell densities were significantly reduced in the brains of the P112 offspring from the established ELS and novel ELS groups than those in the brains of the P112 offspring from the control group (*F*_2,12_ = 39.4, *p* < 0.0001; 5 photographs of 0.58 mm^2^ cortical layer in mice (3 male, 2 female) for each group; Figure 5b,e). Additionally, the cell densities of cortical Iba-1^+^ in the brains of P112 offspring from the established ELS and novel ELS groups were significantly higher than those in the brains of P112 offspring from the control group (*F*_2,12_ = 32.96, *p* < 0.0001; 5 photographs of 0.58 mm^2^ cortical layer in mice (3 male, 2 female) for each group; Figure 5c,f). The novel ELS groups showed markedly higher cell densities of cortical Iba-1^+^ in the brains of P112 offspring mice than in the established ELS group (Figure 5c,f). These results indicate that the novel ELS model downregulated the cell distribution of GABAergic interneurons and induced microglia reactivity in the brains of offspring mice compared to the established ELS model.

## 3. Discussion

During the lactation period, the interaction between infants and their dams supplies most of the requirements for brain development, survival, and growth [14,30]. Disturbances in the relationship between offspring and their dams may lead to stress responses during the postnatal period [31,32]. The correlation between maternal care and stress response in newborn infants has been previously reported [32,33,34]. Previous studies have demonstrated that the stress response induced during the postnatal period may affect brain function, neuroplasticity, neurodevelopment, and behavioral reactions [6,35].

To investigate the adverse effects of stress on brain health in the postnatal period of offspring, many researchers have used ELS models that apply the maternal separation paradigm, limited bedding material, and mesh platform conditions [36]. In the chronic ELS mouse model, ELS resulted in the disruption of maternal interaction and elevated basal plasma corticosterone levels, indicating induction of the stress response and memory impairment in the offspring mice [37]. Additionally, ELS delayed hippocampal development in P10 mice and reduced the number of hippocampal stem cells in P163 adult offspring mice [38]. In this study, we built a novel ELS model that combines diverse ELS conditions, such as the maternal separation paradigm, limited bedding material, and mesh platform conditions, thereby enabling a detailed study of the impacts of ELS on brain development. The novel ELS model triggered body and brain weight reduction; however, this was reversed in P112 mice. In addition, we found that the novel ELS model upregulated *AVP* gene expression in the brains of P14 offspring mice. Johnson et al. reported that ELS induced a reduction in body weight in P14 and P28 mice, but weight loss by ELS displayed a tendency to recover in P60 mice—the adult stage [39]. It was demonstrated that the brain weight change was not induced by the maternal separation model, which was similar to the established ELS model we applied [40]. In addition, the maternal separation paradigm did not induce activation of *AVP* expression in P15 mice brains [41]. These correspond with our results, and our results suggest that the novel ELS model may not only adversely affect mouse brain development in the early postnatal period, but may also be considered a more stressful condition than the established ELS model.

Previous studies have suggested that early life adversity may predispose individuals to psychological disorders, such as depression, anxiety disorder, and anhedonia [42,43,44]. In a comparative research based on humans, women who suffered from abuse showed higher susceptibility to postpartum depression than women who did not [33]. A previous study reported that children who experienced early life adversity, such as physical abuse, violence, and parental absence, showed a higher incidence rate of anxiety disorder and depression in adulthood than children who did not [45]. Recent research on experimental animals has indicated that ELS induces anxiety-like behavior by enhancing neuronal excitability in the basolateral amygdala projection neurons [46]. The offspring mice that suffered from ELS showed impairments in social novelty behavior, but no memory impairments [47]. In addition, offspring rats suffering from ELS showed a reduction in body weight, as well as anxiety- and depression-like behavior [48]. In this study, the novel ELS model induced anxiety- and depression-like behavior in offspring mice. Moreover, the novel ELS model caused social deficiency and memory dysfunction in the offspring mice. This study demonstrated that depression-like behavior and memory impairments induced by the novel ELS model were more negative than the behavioral changes induced by the established ELS model. It was previously reported that the maternal separation paradigm induced memory dysfunction in the Morris water maze test; however, our study showed that the maternal separation paradigm (the established ELS model) tended to decrease memory function in the test trial of the Morris water maze test, but the decrease was not significant [49,50]. This was considered a result due to the relatively small number of animals subjected to the Morris water maze test compared to other behavioral experiments in this study. Collectively, these results suggest that the negative effects of ELS may differ in accordance with the conditions of ELS, and that the novel ELS model may cause more negative behavioral phenotypes than the established ELS model in offspring mice. Therefore, the novel ELS model may be more appropriate than the established ELS model for the in-depth study of behavioral responses caused by ELS.

GABAergic interneurons regulate neural circuits and circuit activity by controlling the activity of principal neurons in the central nervous system and are known to contain calcium-binding proteins, such as CaBP-28k and PV, and neurotransmitters like Vip. [51,52,53,54]. In GABAergic interneurons, CaBP-28k and PV act as buffers for the maintenance of intracellular calcium ion concentration and contribute to the activation of calcium-dependent signaling [55,56]. In addition, Goff et al. reported that Vip is an important mediator that regulates neural dynamics in the brain, and that Vip interneuron dysfunction may cause neurodevelopmental disorders [57]. Several studies using the ELS model have found that ELS-induced changes in the expression of CaBP-28k, PV, and Vip are associated with behavioral alterations in mice. It has been demonstrated that downregulation of CaBP-28k levels, mediated by the ELS-induced elevated corticotropin-releasing hormone receptor type 1 pathway, contributes to memory impairment in the hippocampus of offspring mice [58]. Mouse brains, in which the expression of CaBP-28k was reduced by antisense transgenesis, induced dysfunction of long-term potentiation in the CA1 hippocampal region and memory deficit [59]. In juvenile female rats, ELS causes a reduction in PV protein levels in the prefrontal cortex and induces social deficits [23]. Lussier et al. reported that the reduction of PV+ GABAergic interneuron in the medial prefrontal cortex and hippocampus by prenatal stress leads to social deficits and anxiety behavior in adult mice [60]. It was reported that Methyl-CpG binding protein 2 deletion in Vip interneurons causes disturbance in cortical activity and dysfunction of social behavior [61]. In the dorsolateral prefrontal cortex of postmortem schizophrenia patients, the mRNA levels of *PV* and *Vip* were decreased compared with those of normal humans [62]. In the present study, the novel ELS model decreased *PV*, *CaBP-28k,* and *Vip* gene expression more than the established ELS model in the brains of P14 offspring mice. We found that the novel ELS model induced a more significant reduction in CaBP-28k^+^ cell numbers in the brains of offspring mice than the established ELS model. Additionally, the PV^+^ cell number was significantly reduced in the brains of the novel ELS group compared to that in the brains of the control group. These results indicate that the novel ELS model may affect GABAergic interneuron maintenance more negatively than the established ELS model and exacerbate abnormal behavioral phenotypes. In future studies, we will investigate whether the novel ELS model specifically impairs medial and caudal ganglionic eminences, the origin regions of PV and Vip interneurons [63]. Additionally, we will study the molecular mechanism associated with the downregulation of the GABAergic interneuron marker genes by a novel ELS model because the exact molecular mechanism was not investigated in this study.

In this study, we found that the novel ELS model significantly increased the number of Iba-1^+^ cells, indicating increased microglia reactivity in the brains of the offspring mice. In the central nervous system, microglia are the main players of the innate immune system and are involved in the maintenance of normal brain functions, as well as immune surveillance in the brain [64,65]. Microglia, activated by damaged neurons and infectious agents, induce inflammatory effects by releasing cytokines, such as interferon-γ and interleukin (IL)-8, and phagocytosing various materials, such as cellular debris and apoptotic cells [66,67]. Previous studies have shown that the ELS induces immune responses through microglia reactivity in the brain. It has also been reported that ELS increases the number of Iba-1^+^ cells in the dorsal striatum and the mRNA level of *IL6* in the hippocampus [24]. Moreover, ELS increased the phagocytic activity of microglia and induced the expression of pro-inflammatory genes, such as *IL6*, *IL27*, and *Tnfrsf13b*, in the hippocampus of P28 mice [68]. Studies with molecular mechanisms, however, are needed to further confirm that the novel ELS model induces microglia reactivity. Collectively, these results suggest that the novel ELS model described in this study may induce immune responses more actively in the brains of offspring mice than the established ELS model, and is therefore appropriate for investigating the exact molecular mechanisms of ELS-induced neuroinflammation.

## 4. Materials and Methods

### 4.1. Animals

Specific pathogen-free adult C57BL/6J male and female mice (8 weeks old, 25–30 g) were obtained from Samtaco (Osan, Gyeonggi, Republic of Korea). Mice were group-housed and maintained for time-controlled breeding in standard cages. Mice were kept at 12/12 h light–dark cycle (lights on at 7 a.m.), water and food ad libitum, and conditioned rooms (22 °C, humidity 30%). After acclimatization, the female mice were mated with adult male mice overnight in a ratio of 2:1; subsequently, the day on which a vaginal plug was observed was considered as embryonic day (E) 0.5. The pregnant mice were only present in the home cage, individually, from gestation through weaning (P28.5). The dams were randomly divided into three groups: control, established ELS, and novel ELS (n = 4 dams/control group, n = 2 dam/established ELS group, n = 3 dams/novel ELS group, and 1 mouse per cage). After weaning (P28.5), the female and male offspring were separated and housed in groups of 3–5 animals until P112 (n = 26 (13 males, 13 females) for controls, n = 14 (6 males, 8 females) for established ELS, and n = 22 (10 males, 12 females) for novel ELS). To compare the effects of the established and novel ELS models on the brain development of early postnatal mice, the maternal mice were divided into three groups: control, established ELS, and novel ELS groups (n = 1 dam/group). On P14, the offspring mice from all groups were sacrificed (n = 8 (5 males, 3 females) for controls, n = 6 (3 males, 3 females) for established ELS, and n = 6 (2 males, 4 females) for novel ELS). The litter size per dam for each group, used for all experiments, is stated in Table S1. The Institutional Animal Care and Use Committee of Pusan National University approved all experimental protocols, and all experiments were conducted in accordance with the ARRIVE guidelines and the ILAR Guide to the Care and Use of Experimental Animals.

### 4.2. Early Life Stress Paradigm

**Figure 6:**
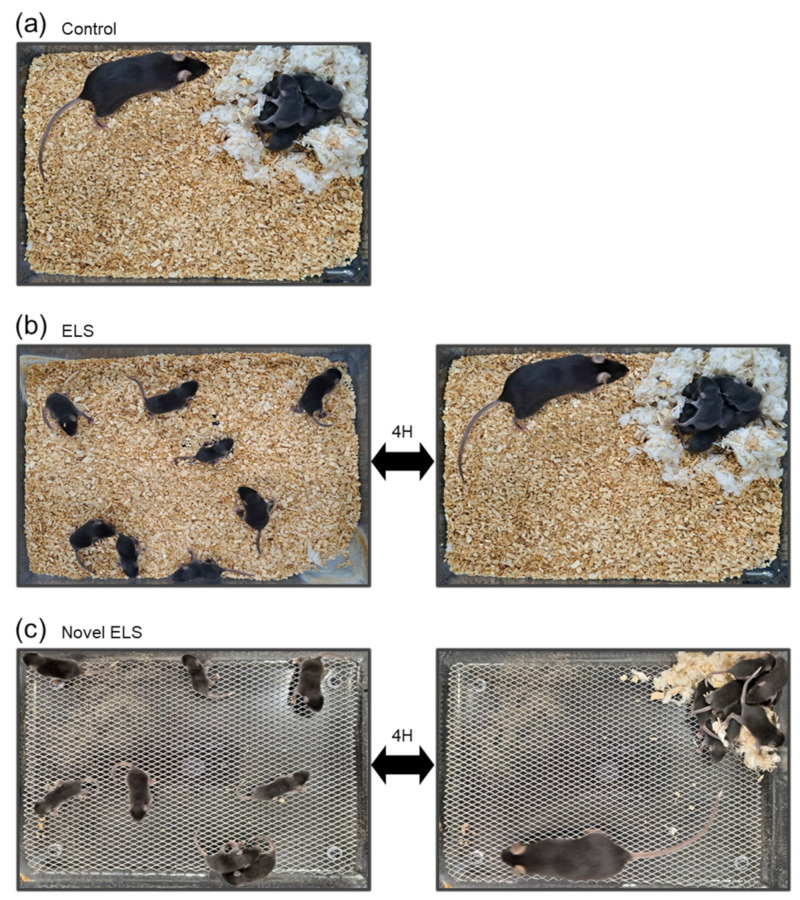
Photographs showing the setup of the early life stress (ELS) paradigm as compared to the control group cage. (**a**) The cage of the control group with a standard amount of sawdust bedding. (**b**) The cage of the established ELS group with a standard amount of sawdust bedding for the maternal separation paradigm. (**c**) The cage of the novel ELS group with a fine-gauge aluminum mesh platform without sawdust bedding.

Dams were monitored every 12 h to check for signs of birth. The day of parturition was designated P0. From P2 onwards, pups were assigned to either the control, established ELS, or novel ELS groups. The control group was provided with 400 mL of standard sawdust bedding and a sufficient amount of nesting material (4.8 g of Nestlet, Indulab, Gams, Switzerland). The control group’s mice were bred in maternity cages with their dams, without any interference (Figure 6a). The established ELS condition, as a maternal separation paradigm, was designed based on an established protocol, with minor modifications [69]. Additionally, we selected 2 weeks as a stress-inducing period based on previous research about the impact of ELS on brain health [13,70,71,72]. The mice of the established ELS group were separated from their dams for 4 h (11:00–15:00) daily, from P2 to P14, in a novel cage with 400 mL of standard sawdust bedding (Figure 6b). After 4 h of separation, the pups were returned to their home cages that contained their dams and were bred in the same conditions as that of the control group. The novel ELS group underwent a maternal separation paradigm in which pups and their dams were separated for 4 h (11:00–15:00) daily, from P2 to P14, in a novel cage with a fine-gauge aluminum mesh platform, unlike that applied for the established ELS group. The mice in the novel ELS group were bred from P2 to P14 in their home cage, which contained a fine-gauge aluminum mesh platform with no sawdust bedding and a limited amount of nesting material (2.4 g of Nestlet; Figure 6c).

### 4.3. Behavioral Analysis

Experimental design: at 9 weeks of age, offspring mice were selected randomly for behavioral tests, as previously described [73,74]. All behavioral tests were conducted during the light cycle. On the testing days, mice were transferred to the testing room for at least 30 min before test commencement, and testing was conducted by laboratory technicians who were blinded to the mouse group information. All experiments were conducted between 8:00 a.m. and 2:00 p.m. and a resting period of 2 days per 1 week was provided between two consecutive tests. All the experimental areas were cleaned using 70% ethanol before conducting the tests and between each test.

#### 4.3.1. Tail Suspension Test

Each mouse was suspended by attaching (with adhesive tape) the tail to the edge of a shelf 50 cm above the surface of a table. The mice were allowed to move for 6 min, and their behavior was recorded with a camera. The videos were analyzed using EthoVision^®^ XT16. The immobility time was recorded over the last 5 min of the experiment.

#### 4.3.2. Forced Swimming Test

Each mouse was gently placed in a glass cylinder (20 cm in height and 15 cm in diameter) filled with water (25 °C ± 2 °C), to a depth of 12 cm. For the pre-test, all mice underwent water exposure for 15 min. After 24 h, all mice were forced to swim for 5 min, and the duration of immobility was recorded by the camera. The videos were analyzed using EthoVision^®^ XT16.

#### 4.3.3. Elevated Plus-Maze Test

The elevated plus-maze test was performed, as previously described [74]. The apparatus included two open arms (35 × 5 cm), two enclosed arms (35 × 5 × 15 cm), and a central platform (5 × 5 cm). The apparatus was elevated 45 cm above the floor. The mouse was placed on the central platform facing the open arms and allowed to roam freely for 5 min. The percentage of time in the arms was calculated as per the following formula: (time spent in open arms/time spent in total arms) × 100.

#### 4.3.4. Open-Field Test

The open-field test was performed on a large 50 cm tall and 60 cm wide acrylic cube with a white bottom. Briefly, to evaluate locomotor activity, mice were individually placed near the wall and allowed to move freely for 5 min. The movement of the mice was recorded and analyzed using the EthoVision^®^ XT16 software (Noldus, Leesburg, VA, USA). The time spent in the center zone, frequency of entry into the center zone (15 × 15 cm imaginary square), velocity, and distance traveled were measured and documented.

#### 4.3.5. Social Interaction Test

The experimental mouse and stranger C57BL/6J mouse were introduced into the open field from opposite sides of the apparatus and were allowed to explore it freely for 10 min. The interaction indices, including general sniffing, anogenital sniffing, and following, were counted manually.

#### 4.3.6. Three-Chamber Social Test

The three-chambered apparatus was composed of three Plexiglas chambers, each measuring 20 × 40 × 22 cm, and dividing walls with small square entrances (10 × 5 cm) that allowed free movement to each chamber. Both side chambers contained a cylindrical plastic cage (17 cm in height and a bottom diameter of 8 cm, with bars spaced 1 cm apart) in the corner that was used to confine the stranger mice. The three-chambered social test was then executed according to a previous study [75]. First, the subject mice were placed in the apparatus to freely explore all three chambers, with an empty plastic cage in each side chamber (5 min habituation period). For sociability testing, an unfamiliar C57BL/6J mouse (Stranger 1, “S1”) was confined in the cylindric plastic cage in one of the side chambers, and an empty cylindrical plastic cage (Empty “E”) was kept on the other side of the chamber. The subject mouse was then placed in the center chamber and allowed to freely explore all three chambers for 10 min. For the social novelty test, the empty plastic cage was replaced with another unfamiliar C57BL/6J mouse (Stranger 2, “S2”) and the subject mouse was again allowed to freely explore all the three chambers for 10 min. All stranger mice were of the same sex and age as the subject mice and were habituated to plastic cages. The time spent in the chamber, distance traveled, and heat-maps were measured using EthoVision^®^ XT16 software.

#### 4.3.7. Morris Water Maze

The Morris water maze test was performed in a circular pool (90 cm in diameter and 40 cm in height) filled with water (25 °C ± 1 °C). The water was made opaque by the addition of skim milk. The tank was divided into four equal sections (I, II, III, and IV). A circular platform (10 cm in diameter and 20 cm in height) made of Plexiglass was placed in the middle of the target quadrant (III; 12 cm from the edge of the pool and 1 cm below the surface of the water), with visual cues on the pool walls as spatial references. The mice received a two-phase training protocol for 7 days, comprising cue training for 3 days, followed by spatial training for 4 days. Four trials were conducted per mouse daily and the escape latency (time to locate the hidden platform) in each trial was recorded. For each trial, the subject mice were gently placed into the water facing the wall from one of three quadrants (I, II, and IV), which varied according to the day of testing. The mice were then given 1 min to locate the platform, and the trial was completed when the mice had located the platform. If the mouse failed to locate the platform within the 1 min period, it was gently guided onto the platform and allowed to rest for 30 s. The mean of the escape latencies (s) for the four trials is represented as the learning result for each mouse (training section). On day 8 (probe test day), the platform was removed from the pool and the mice were allowed to search for it for 60 s. The videos were recorded and analyzed using EthoVision^®^ XT16 software. The amount of time spent on the target platform, number of crossings performed on the platform, distance, and velocity were measured to determine the memory results.

#### 4.3.8. Novel Object Recognition

First, the subject mice were placed in the open-field arena in the presence of two identical objects (2 × 5 × 9 cm) and were allowed to explore the open-field arena freely for 10 min. After 6 h, one of the objects was replaced by a novel object of different shape and color compared to the old object; the subject mice were again placed inside the open-field arena to freely explore it for another 10 min. The amount of time that the mice spent with the novel and old objects (sniffing or exploring at a distance within 2 cm of the object) was recorded. The recognition index was calculated as: (amount of time spent interacting with the novel object)/(amount of time spent with both the novel and old objects) × 100. The videos were analyzed using EthoVision^®^ XT16 software.

### 4.4. RNA Extraction and Quantitative Real-Time PCR

Total RNA was extracted from the whole brain of each mouse using the TRIzol reagent (Ambion, Austin, TX, USA), according to the manufacturer’s protocols. Extracted RNA was quantified using an Epoch microplate spectrophotometer (BioTek, Winooski, VT, USA). RNA purity was estimated by A260:A280 ratios. Subsequently, 1 μg of RNA was used for cDNA synthesis, and cDNA synthesis was performed using M-MLV reverse transcriptase (Invitrogen, Carlsbad, CA, USA), according to a previously described protocol [76]. Real-time PCR was performed using a SYBR Green system (Applied Biosystems, Foster City, CA, USA). Quantitative real-time PCR was performed using QuantStudio 3 (Applied Biosystems, Foster City, CA, USA). GAPDH served as an internal control. The expression of the target gene was normalized to the expression of the GAPDH gene by using the 2^−ΔΔCt^ method [77]. Relative expression of the target gene in the established, novel ELS groups was calculated based on the gene expression of the control group as 100%. The primer sequences are as follows: *AVP* (F: 5′-TCGCCAGGATGCTCAACAC-3′; R: 5′-TTGGTCCGAAGCAGCGTC-3′), *Pvalb* (F: 5′-AGCCTTTGCTGCTGCAGACT-3′; R: 5′-GGCCCACCATCTGGAAGAA-3′), *Vip* (F: 5′-GCAGCAGCATCTCGGAAGAT-3′; R: 5′-TGTGAAGACGGCATCAGAGTGT-3′), *CaBP-28k* (F: 5′-GACGGAAAGCTGGAAACTGGAACTGAC-3′; R: 5′-AGCAAAGCATCCAGCTCATT-3′).

### 4.5. Immunofluorescence

Mice were anesthetized with avertin (2,2,2-tribromoethanol: T48402, Sigma-Aldrich, Burlington, MA, USA; ter-amyl alcohol: 240486, Sigma-Aldrich, USA; 0.018 mL (2.5%) per gram of body weight) before they were sacrificed. The extracted brains were briefly fixed in phosphate-buffered 4% paraformaldehyde at 4°C. They were transferred to 1x phosphate-buffered saline (PBS) and embedded in agarose. The embedded brain was sectioned coronally at 80 μm with a vibratome (Leica, VT1000S). The brain sections were permeabilized using PBS containing Triton™ X-100 (Sigma-Aldrich, USA) (0.5% for tissues). The tissues were then blocked using blocking buffer (PBS + 5% goat serum (Vector Laboratories, Burlingame, CA, USA) + 0.25% Triton™ X-100) for 1 h, followed by overnight incubation at 4 °C with primary antibodies against PV (catalog no. MAB1572, 1:500, Sigma-Aldrich, USA; CaBP-28k, catalog no. ABN2192, 1:1000, Millipore, Burlington, MA, USA; Iba-1, catalog no. 019-19741, 1:500, Wako, Richmond, VA, USA). For secondary staining, tissues were incubated for 1 h with a secondary antibody solution (Alexa Fluor™ 488 goat anti-mouse IgG, catalog no. A11001, 1:1000; Alexa Fluor™ 488 goat anti-rabbit IgG, catalog no. A11034, 1:1000, Invitrogen, Carlsbad, CA, USA) containing 100 ng/mL 4′,6-diamidino-2-phenylindole (Sigma-Aldrich, USA). The cells were then mounted in Fluoro-Gel (Emsdiasum, Hatfield, PA, USA). Fluorescently labeled cells in the brain tissue were visualized by a fluorescence microscope (Axio Observer 7 with Apotome 3; Zeiss, Oberkochen, Germany) and were quantified by Image J software [78].

### 4.6. Statistical Analysis

All statistical analyses were conducted using a one-way ANOVA for more than two independent groups (Bonferroni’s multiple comparison test for comparing all pairs of columns) and an unpaired Student’s *t*-test (for comparing two independent groups). Data were collected randomly and analyzed using Prism software (GraphPad 5, San Diego, CA, USA). The results are presented as mean ± SEM, and the *p*-values for each comparison are described in the Results section. Each experiment was performed in a blinded and randomized manner. Animals were randomly assigned to different experimental groups, and data were collected and processed randomly. The allocation, treatment, and handling of the animals were similar across the study groups. The results of all ELS groups were compared with the results of the control group. All experiments were independently performed in triplicate.

## 5. Conclusions

The novel ELS model was more effective in inducing anxiety-like behavior and promoting depression-like behavior than the established ELS model. Offspring mice exposed to the novel ELS model showed social deficits and more severe memory dysfunction than those exposed to the established ELS model. The novel ELS model induced a disturbance in the growth of the mice and altered the gene expression related to stress response and GABAergic interneurons in the brains of P14 offspring mice. The novel ELS model caused a decrease in the number of cortical GABAergic interneurons and induced microglia reactivity in the brains of adult offspring mice. Taken together, this novel ELS model of early postnatal manipulation will be able to contribute to a clearer understanding of the negative effects of ELS on brain health.

## Data Availability

The data used to support the findings of this study are available from the corresponding author upon request.

## Supplementary Materials

The following supporting information can be downloaded at: https://www.mdpi.com/article/10.3390/ijms24054688/s1.
