# Supplementary material for: A Novel Early Life Stress Model Affects Brain Development and Behavior in Mice
Source: Int J Mol Sci. 2023 Feb 28;24(5):4688. doi: 10.3390/ijms24054688 (PMC10002977; doi:10.3390/ijms24054688)
Supplement: Supplementary file 1 [file ijms-24-04688-s001.zip › ijms-2197894 - supplementary.pdf]

Supplementary information

A Novel Early Life Stress Model Affects Brain Development and Behavior in Mice

Hyun Seung Shin, Soo Min Choi, Seung Hyun Lee, Ha Jung Moon and Eui-Man Jung*

Department of Molecular Biology, College of Natural Sciences, Pusan National University, Busan, 46241, Republic of Korea

* Corresponding author

Running Title: Effects of novel ELS model on neurodevelopment.

Keywords: Early life stress, Behavior, Mice, Calbindin-D_{28k}, Parvalbumin

Correspondence: Prof. Eui-Man Jung

Department of Molecular Biology, College of Natural Sciences,

Pusan National University, Busan, 46241, Republic of Korea

Tel: +82-51-510-3682; Fax: +82- 51-513-9258; E-mail: jungem@pusan.ac.kr

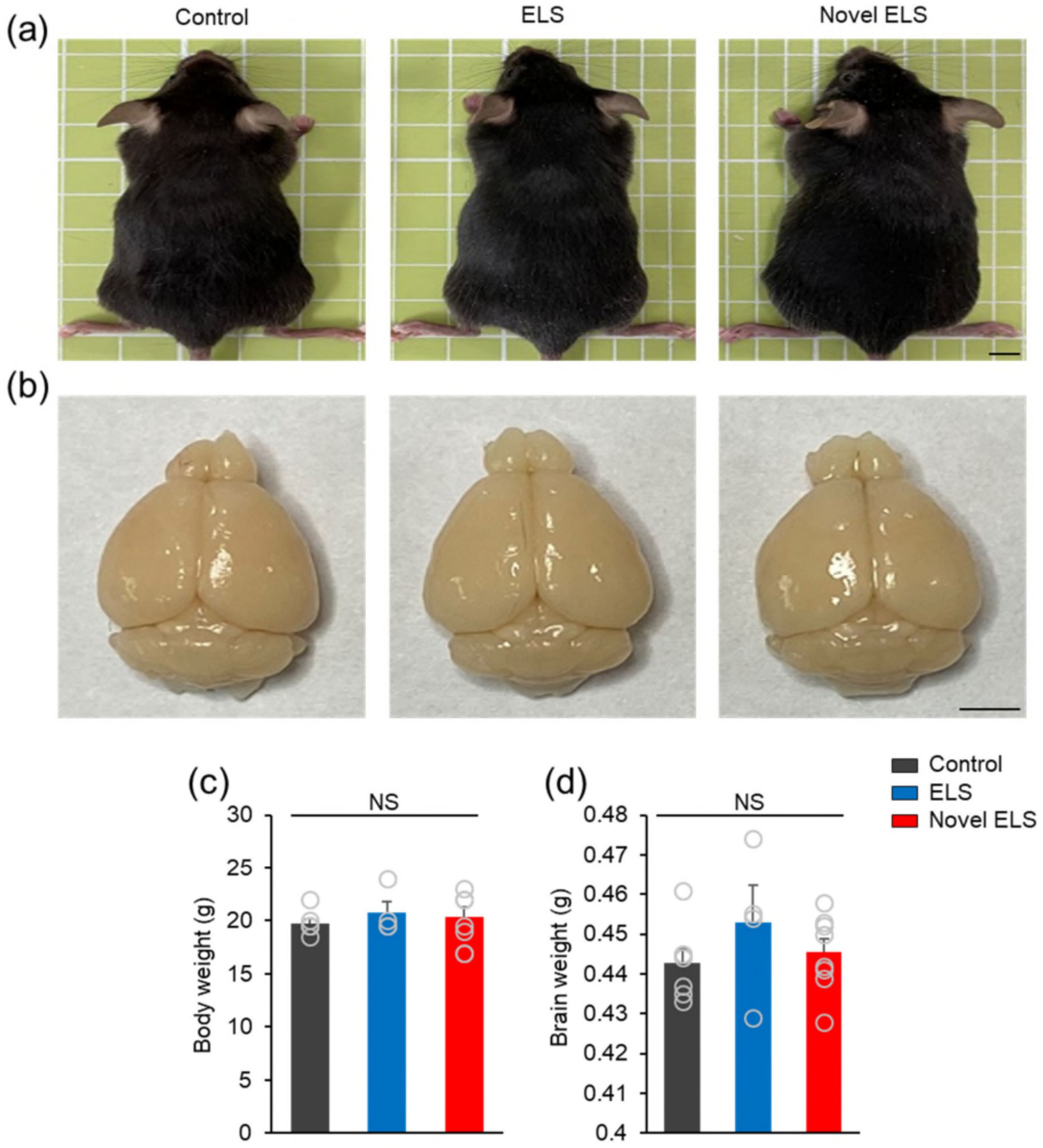

Figure S1. The impact of novel ELS on mouse growth does not last until the adult stage. (a, b) Representative image of the P112 mouse body and brain for the control, established ELS, and novel ELS groups. (c, d) P112 offspring mouse body and brain weight. Scale bar = 5 mm (body image), 3 mm (brain image). Statistical significance was determined using one-way ANOVA, with Bonferroni's correction. NS means no signification.

Table S1. The litter size per dam for each group.

Group \ Dam	1	2	3	4	5
Control	n=5 (M: 3, F: 2)	n=7 (M: 2, F: 5)	n=5 (M: 3, F: 2)	n=9 (M: 5, F: 4)	n=8 (M: 5, F: 3)
Established ELS	n=8 (M: 4, F: 4)	n=6 (M: 2, F: 4)	n=6 (M: 3, F: 3)	-	-
Novel ELS	n=6 (M: 2, F: 4)	n=8 (M: 3, F: 5)	n=8 (M: 5, F: 3)	n=6 (M: 2, F: 4)	-
